# Complete genome sequence of the biopesticidal *Burkholderia ambifaria* strain BCC0191

**DOI:** 10.1128/mra.01097-24

**Published:** 2024-12-12

**Authors:** Gordon Webster, Alex J. Mullins, Eshwar Mahenthiralingam

**Affiliations:** 1 Microbiomes, Microbes and Informatics Group, Organisms and Environment Division, School of Biosciences, Cardiff University, Cardiff, Wales, United Kingdom; Rochester Institute of Technology, Rochester, New York, USA

**Keywords:** *Burkholderia*, biocontrol, genome mining, cepacin

## Abstract

Here, we report the genome sequence of *Burkholderia ambifaria* BCC0191, a biopesticide originally isolated from the barley rhizosphere. The genome was assembled using an Illumina–Nanopore hybrid approach and consisted of 7.62 Mbp distributed across three replicons. Several specialized metabolite biosynthetic gene clusters, including those known to be active in biocontrol, were identified.

## ANNOUNCEMENT

*Burkholderia ambifaria* (1) is a member of the *Burkholderia cepacia* complex (Bcc), a group of closely related species found in soil, water, and rhizosphere. Some Bcc species protect plants from disease, promote plant growth, and cause opportunistic infection in immunocompromised individuals, including those with cystic fibrosis (2). However, *B. ambifaria* is rarely encountered in such infections (3), with none found in a 2017 UK survey (4). *B. ambi*faria BCC0191 (5), originally isolated as strain J82 (alternatively named ATCC 51993 or ARS BcB) from the rhizosphere of greenhouse-grown barley in soil from a Wisconsin cornfield, was shown to have significant antifungal activity (6). Subsequently, strain J82 was registered by the United States Environmental Protection Agency as a biopesticide and used commercially in various formulations (e.g., Blue Circle), before being withdrawn due to potential risks to human health (7). The recent findings that *B. ambifaria* BCC0191 can protect pea seedlings from oomycete damping-off and did not cause disease in a murine respiratory infection model (5) have sparked resurgence in its potential as a biopesticide (8).

**Fig 1:**
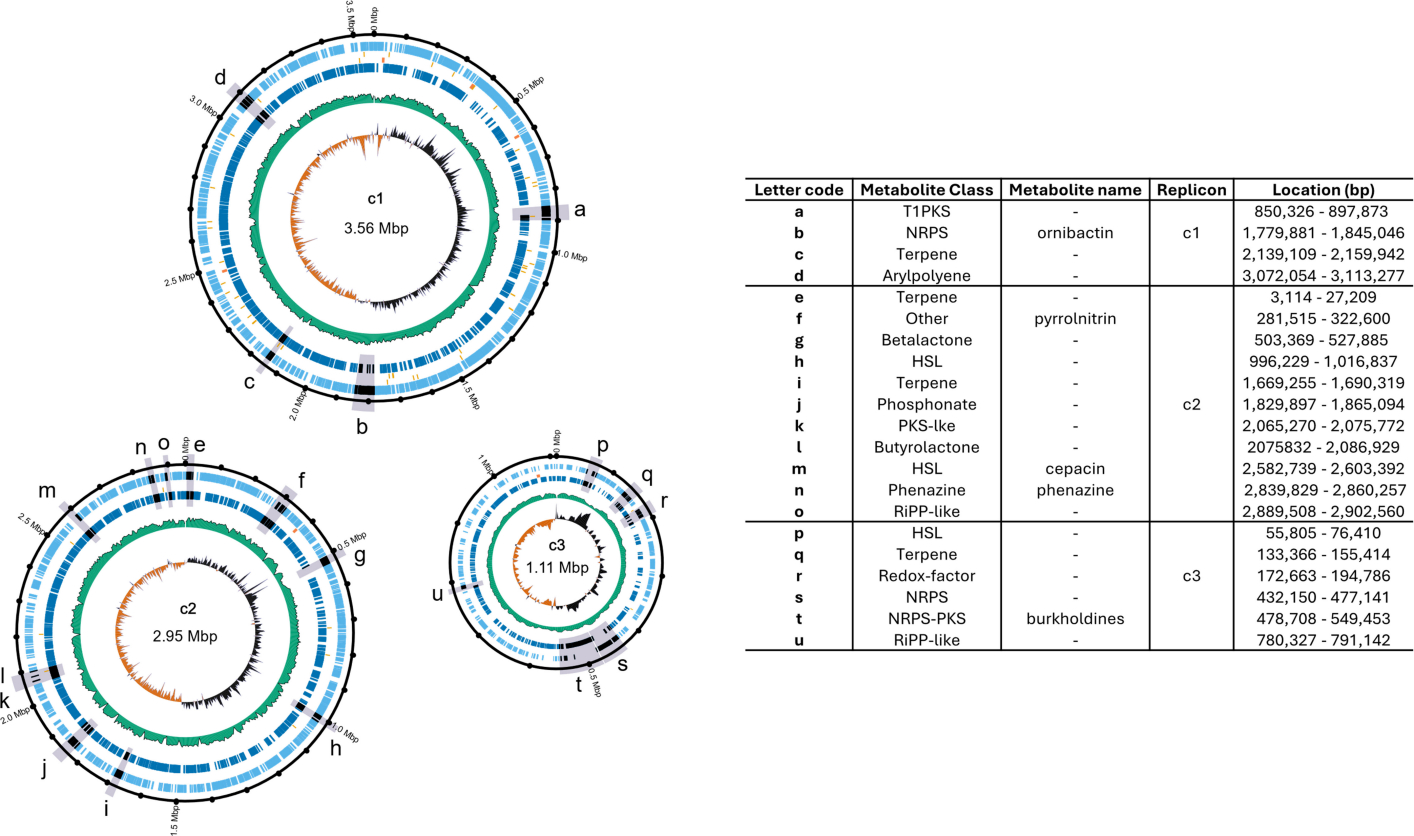
Genomic map and table of specialized metabolite biosynthetic gene clusters (BGCs) of *Burkholderia ambifaria* BCC0191. Genomic map of *B. ambifaria* created using GenoVi version 0.4.3 (14); inner to outer ring: GC skew, GC content, rRNA genes, tRNA genes, negative strand CDS, and positive strand CDS. Positions of BGCs are indicated by letters and shaded wedges. The table shows details of BGC metabolite class, replicon location, base pair position, and named characterized metabolites as predicted by antiSMASH.

Strain BCC0191 is routinely cultured on tryptone soya broth (TSB) and stored in TSB with 8% dimethyl sulfoxide at −80°C. For genome sequencing, BCC0191 was grown in 5 mL of TSB at 30°C overnight at 50 rpm. Cells were harvested by centrifugation, and gDNA was extracted using a Maxwell 16 Instrument and Tissue DNA purification Kit (Promega) according to the manufacturer’s instructions. Fragment size and concentration were assessed using an Agilent Tapestation and Qubit 3 fluorometer. Approximately 15 µg of gDNA was sheared to 20 kbp using the Covaris g-TUBE, and size exclusion was performed with AMPure XP beads (Beckman Coulter) to remove fragments <1 kbp. DNA was eluted in 20 µL of molecular-grade water. A long-read sequencing library was generated using a rapid barcoding sequencing kit (SQK-RBK004) and sequenced on a MinION (MIN-101B) device, using the FLO-MINSP6 R9.4.1 flow cells (ONT). Raw data reads were acquired using MinKNOW software (ONT), trimmed and de-multiplexed with Porechop v0.2.4 (9), and further corrections performed using Canu v1.8 (10) under default settings. Hybrid genome assembly was constructed using Unicycler v0.4.7 (11) with previously published (12) Illumina reads of BCC0191 (ERS784799) and scaffolded with corrected MinION reads using default settings (119× genome coverage). The polished genome assembled into three genomic replicons, c1, c2, and c3 (Fig. 1), and each replicon was reorientated using Circlator v1.5.5 (13) at the *dnaA*, *parA*, and *parB* gene start positions, respectively. The genome assembly was annotated with Prokka v1.14.6, and the genome size and other metrics are as follows: 7.62 Mbp, three replicons, 66.5 guanine–cytosine (GC), 6,633 predicted coding sequence (CDS), 6,729 predicted genes, 18 rRNA, and 77 tRNA genes.

Specialized metabolite biosynthetic gene clusters (BGCs) within *B. ambifaria* BCC0191 were identified by genome mining using antiSMASH v6.1.1 (15). The antiSMASH results predicted 21 BGCs encompassing 14 metabolite classes (Fig. 1). BGCs included the known antimicrobial compounds cepacin, pyrrolnitrin, phenazine, and burkholdines (NRPS-PKS), and the siderophore ornibactin (NRPS). Uncharacterized BGCs included one further NRPS, two PKS, two RiPP-like, one phosphonate, and four terpene clusters, among others (Fig. 1). These characterized antimicrobials, especially cepacin (5, 8), are known to contribute to biopesticidal activity of *B. ambifaria* BCC0191.

## Data Availability

The genome sequence in this announcement has been deposited in NCBI GenBank under the BioProject accession number PRJNA1035503 and genome assembly accession number GCA_043193125. The Illumina paired-end read data associated with this genome (BioSample accession number ERS784799) was previously deposited under the BioProject accession number PRJEB9765 and short read archive (SRA) accession ERX1188530.

## AFTER EPUB

*[This article was published on 12 December 2024 with an error in the Data Availability. The last accession number link was corrected in the current version, posted on 20 December 2024.]*
